# Creatine supplementation and endurance performance: surges and sprints to win the race

**DOI:** 10.1080/15502783.2023.2204071

**Published:** 2023-04-25

**Authors:** Scott C. Forbes, Darren G. Candow, Joao Henrique Falk Neto, Michael D. Kennedy, Jennifer L. Forbes, Marco Machado, Erik Bustillo, Jose Gomez-Lopez, Andres Zapata, Jose Antonio

**Affiliations:** a Brandon University, Department of Physical Education Studies, Brandon, MB, Canada; b University of Regina, Faculty of Kinesiology and Health Studies, Regina, SK, Canada; c University of Alberta, Faculty of Kinesiology, Sport, and Recreation, Edmonton, AB, Canada; d Universiade Iguaçu Campus V, Itaperuna, RJ, Brazil; e Train 8Nine/CrossFit Coconut Grove, Erik Bustillo Consulting, Miami, FL, USA; f Rehab & Nutrition Center, Human Performance Laboratory, Motion Training, Lo Barnechea, Chile; g Motion Sports Nutrition, Medellín, Colombia; h Nova Southeastern University, Department of Health and Human Performance, Davie, FL, USA

**Keywords:** Ergogenic aids, supplements, aerobic, anaerobic, exercise

## Abstract

Creatine supplementation is an effective ergogenic aid to augment resistance training and improve intense, short duration, intermittent performance. The effects on endurance performance are less known. The purpose of this brief narrative review is to discuss the potential mechanisms of how creatine can affect endurance performance, defined as large muscle mass activities that are cyclical in nature and are >~3 min in duration, and to highlight specific nuances within the literature. Mechanistically, creatine supplementation elevates skeletal muscle phosphocreatine (PCr) stores facilitating a greater capacity to rapidly resynthesize ATP and buffer hydrogen ion accumulation. When co-ingested with carbohydrates, creatine enhances glycogen resynthesis and content, an important fuel to support high-intensity aerobic exercise. In addition, creatine lowers inflammation and oxidative stress and has the potential to increase mitochondrial biogenesis. In contrast, creatine supplementation increases body mass, which may offset the potential positive effects, particularly in weight-bearing activities. Overall, creatine supplementation increases time to exhaustion during high-intensity endurance activities, likely due to increasing anaerobic work capacity. In terms of time trial performances, results are mixed; however, creatine supplementation appears to be more effective at improving performances that require multiple surges in intensity and/or during end spurts, which are often key race-defining moments. Given creatines ability to enhance anaerobic work capacity and performance through repeated surges in intensity, creatine supplementation may be beneficial for sports, such as cross-country skiing, mountain biking, cycling, triathlon, and for short-duration events where end-spurts are critical for performance, such as rowing, kayaking, and track cycling.

## 1. Introduction

Creatine (α-methyl guandino-acetic acid) is a popular dietary supplement purported to enhance exercise performance [1,2]. Creatine is synthesized endogenously primarily in the kidneys, liver, and pancreas via a two-step process involving three amino acids, arginine, glycine, and methionine [3]. In addition, creatine can be obtained exogenously through the diet (e.g. seafood, red meat, and poultry [4]) and/or as a manufactured commercially available dietary supplement [5]. Importantly, it is difficult to saturate creatine stores within muscles through diet alone. As such, creatine supplementation, even in omnivores, elevates resting total creatine levels by ~20% [1,6]. Once creatine is taken up into the blood, ~95% is stored in skeletal muscle, of which ~67% is converted to phosphocreatine (PCr) and ~33% remains as free creatine [3,7]. Phosphocreatine (PCr) combines with adenosine diphosphate (ADP) and is catabolized by creatine kinase to rapidly resynthesize adenosine triphosphate (ATP) [3]. This reaction is reversible during periods of rest or light activity. Approximately, 2% of whole-body creatine stores are lost daily through the non-enzymatic degradation of creatine to creatinine [4,8].

Creatine supplementation has been well established as an ergogenic aid to enhance resistance training adaptations, including gains in muscular power, endurance, and strength [1,9,10]. Creatine supplementation also improves performance in a single bout of intense exercise (e.g. Wingate or 100-m sprint) [1,2] and in repeated efforts (i.e. multiple sprints) [1,2,11] and has been purported as an effective dietary supplement for team sport athletes [2]. However, the impact of creatine on endurance-based exercise is less known. It is generally believed that creatine has either no effect or possibly a detrimental effect on endurance performance or oxidative power (i.e. maximal oxygen uptake) [12]. The perceived reduction in endurance performance may be associated with short-term creatine loading on intracellular and/or total body water retention which leads to gains in body mass [13,14]. This increase in body mass is thought to impair weight bearing (e.g. running) endurance exercise performance [15]. In contrast, creatine supplementation elevates intramuscular PCr, improves buffering capacity, augments glycogen resynthesis, and reduces oxidative stress and inflammation [1,2,16]. These purported benefits may counter the gains in body mass and have a positive effect on enhancing endurance performance and recovery.

Endurance exercise performance is multifactorial. Race dynamics, changes in elevation, course design, and other factors might influence how athletes pace themselves [17]. Thus, endurance races are rarely performed at a steady pace and often multiple surges are performed at maximal or supramaximal intensities. For example, road cycling, cross-country skiing, mountain biking, triathlon, rowing, and team sports, such as soccer, basketball, hockey, or rugby all involve frequent intensity changes. Furthermore, even if the sport requires an even-pace strategy, such as speed skating or the pursuit in track cycling, a “finishing kick” that requires utilization of anaerobic reserves appears to be critical [17]. In team sports that are aerobic in nature, it is also known that more scoring chances are generated in the latter portion of the half or game where the ability to sprint can be attributed to anaerobic reserves. Thus, overall changes in pace and surges during a race or event at maximal or supramaximal intensities can be key determinants of success. In these situations, the athlete’s anaerobic work capacity (W’) [18] and the recruitment of fast twitch glycolytic fibers (i.e. type II) for increased muscular power may be influenced by creatine supplementation [19].

Thus, the purpose of this narrative is to: (1) identify potential mechanisms whereby creatine may influence endurance performance, defined as large muscle mass activities that are cyclical in nature and longer than ~3 min in duration, (2) evaluate the efficacy of creatine supplementation on endurance performance, (3) to discuss limitations in the current evidence that warrants further investigations, and (4) provide practical recommendations for the use of creatine supplementation as an ergogenic aid for those involved in endurance events.

## 2. Potential mechanisms of creatine supplementation that can influence endurance performance

**Figure 1.**
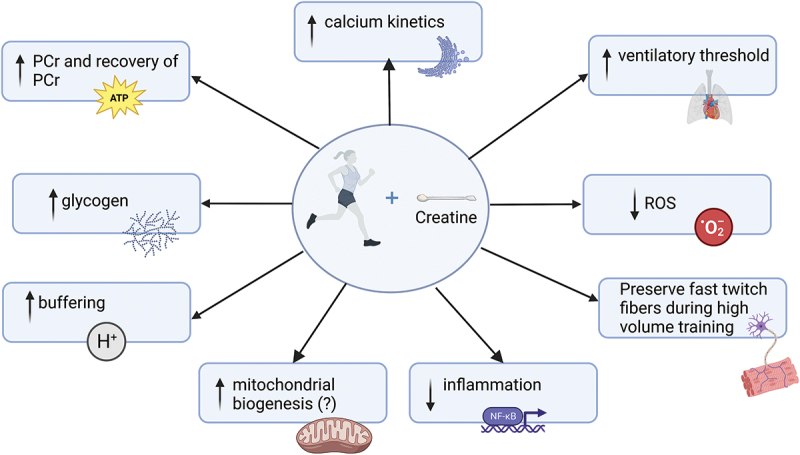
Potential mechanisms of how creatine supplementation can enhance endurance performance. Created with BioRender.Com.

Creatine is a pleiotropic molecule that has been shown to influence several metabolic, hormonal, and physiological factors that may impact endurance performance. The potential mechanisms through which creatine might influence endurance performance are discussed and summarized in Figure 1.

### 2.1. Increases in total creatine and glycogen content

Seminal work by Harris and colleagues in the early 1990s revealed that muscle creatine content can be elevated ~20% following creatine supplementation [20]. Mechanistically, elevated intramuscular PCr and free creatine increase the potential for greater non-oxidative alactic capacity and faster PCr resynthesis during recovery between exercise bouts [1]. The increase in intramuscular concentrations of PCr leads to a greater reliance on the phosphagen system during intense efforts [21] and may be associated with improvements in time to exhaustion [22], repeated sprints [23], anaerobic work capacity [24], and time trial performance [25]. Further, elevated intramuscular creatine stores aid in phosphate shuttling facilitating ATP transport from sites of ATP production (i.e. mitochondria) to sites of ATP utilization [26] which attenuates oxidative stress [27]. Importantly, it should be noted that no form of exercise is purely anaerobic or aerobic, and that all three energy systems (anaerobic alactic, anaerobic glycolytic, and aerobic) contribute to ATP re-synthesis; however, their contribution is determined based on intensity and duration of the exercise.

Creatine supplementation also facilitates the uptake and retention of glycogen [16], possibly through an upregulation and an increase in GLUT-4 content [28] associated with changes in cell volume [29]. Roberts et al. [30] investigated healthy males (*n* = 14) that cycled to exhaustion at 70% VO_2_ peak followed by the ingestion of a high carbohydrate diet (>80% of calories as carbohydrates) with and without creatine (20 g/day). The addition of creatine increased intramuscular creatine stores (total, free creatine and PCr) and muscle glycogen content compared to placebo within 24 h, and these elevated levels were sustained for 6 days. Luc van Loon [29], similarly, found increases in glycogen storage following 5 days of creatine loading (20 g/day), but creatine had no effect on insulin, GLUT-4 mRNA expression, glycogen synthase, or glycogenin-1 mRNA expression. Collectively, these results provide some evidence that creatine can positively enhance muscle glycogen which is an important substrate metabolized during high-intensity or long-duration endurance exercise [31]. Tomcik et al. [32] had 18 elite cyclists ingest creatine (20 g/day for 5 days followed by 3 g/day for 9 days) or placebo with either a high (12 g carbohydrates·kg^−1^) or moderate (6 g carbohydrates·kg^−1^) dose of carbohydrates. Participants performed a 120-km time trial whereby every 10-km’s participants alternated between a 1-km and 4-km sprint that were to be completed as quickly as possible. Creatine ingestion resulted in a significant improvement in power output (relative changes ranged from 1.10 to 1.15 compared to baseline) in the closing sprints. Further, to determine if the gain in body mass from creatine negatively influenced performance, participants completed a time to exhaustion test at an 8% incline on a customized treadmill at a velocity that elicited 90% of VO_2_ peak following the 120-km time trial. The creatine mediated gain in body mass did not have a detrimental effect on performance (creatine: baseline = 5 min 4 s; moderate carbohydrate = 6 min; high carbohydrate = 7 min 17 s; placebo: baseline = 5 min; moderate carbohydrate = 6 min 25 s; high carbohydrate = 6 min 35 s; *P* > 0.05). These authors highlighted that since the ability to stay with the pack during breakaways or sprint to the finish line is key to performance in endurance events, co-ingestion of creatine and carbohydrates appears to be a beneficial strategy in elite cyclists.

### 2.2. Reduced neuromuscular activity and improved buffering capacity

Creatine may affect exercise performance by increasing calcium re-uptake into the sarcoplasmic reticulum, thereby enhancing myofibrillar cross-bridge cycling and force development [1,8]. In addition, creatine acts as an intracellular buffer, since PCr hydrolysis consumes a hydrogen ion [3]. For example, when assessing blood lactate responses to an incremental cycling test (30 W increases every 3 min), creatine supplementation attenuated blood lactate at the end of each stage [21]. This effect was hypothesized to be due to a combination of a reduced reliance on anaerobic glycolysis (due to greater reliance on the phosphagen system) and through an increase in buffering capacity. According to the authors, these changes might indicate that creatine supplementation could be beneficial for athletes who perform multiple surges during a race or during a finishing sprint. Furthermore, in speed endurance events, swimmers (*n* = 19) receiving 10 g/day of creatine (co-ingested with carbohydrates for 7 days) improved their final 50-m sprint during a 400-m race compared to controls [33]. In addition, short-term creatine supplementation (6 g/day for 5 days) enhanced anaerobic performance (sprint intervals) by 18% without impairing endurance performance in triathletes, with no effect on oxygen uptake or blood lactate (assessed after each interval) [25].

**Figure 2.**
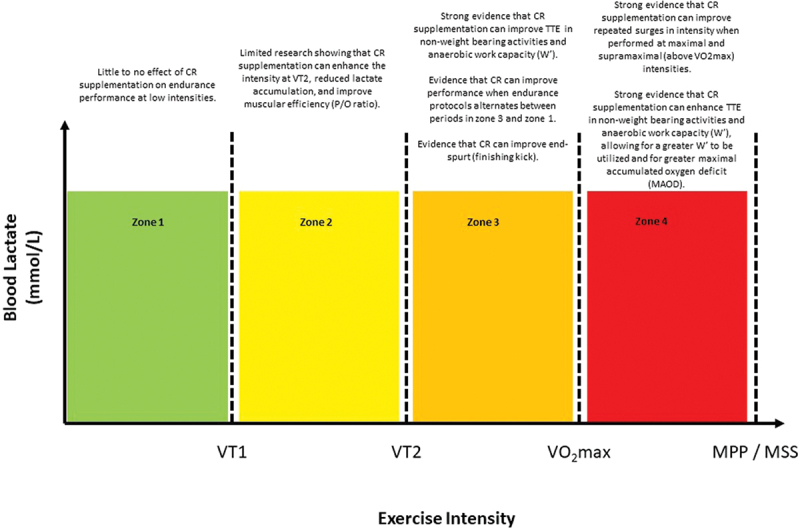
The effects of creatine supplementation to enhance different exercise intensities.

Creatine supplementation also positively effects neuromuscular activity. In a group of recreationally trained female participants [34], creatine (20 g/day for 5 days) increased power output at the electromyography (EMG) fatigue threshold by ~20 W (14.5%). While the exercise bouts were shorter (60 s) than what is traditionally considered to be endurance exercise, the EMG fatigue threshold represents the highest power output that can be sustained for an extended period of time without signs of neuromuscular fatigue [34]. This could, therefore, have implications for endurance exercise that is performed close to, or at the maximal metabolic steady state, since further recruitment of motor units is considered to increase the VO_2_ slow component, contributing to fatigue development [35], as illustrated in Figure 2. Another possible explanation is that creatine increases muscle efficiency (possibly due to calcium kinetics and enhanced force production), reducing motor unit recruitment or the volume of muscle mass that is recruited to perform exercise at a specific intensity [36].

### 2.3. Effects on oxygen consumption, whole-body VO_2_ kinetics, and mitochondrial adaptations

Considering that creatine could, in theory, improve muscular efficiency (i.e. power output to oxygen consumption ratio), creatine supplementation might lead to a lower level of oxygen consumption at a given intensity and enhance whole-body VO_2_ kinetics [36] and increase ventilatory threshold [12], which may be beneficial for endurance performance [37]. Rico-Sanz et al. [38] examined 14 highly trained participants following a creatine loading phase (20 g/day for 5 days) and found significantly lower ammonia and enhanced oxygen uptake when exercising at 90% of maximal power output. Similarly, Jones et al. [36] using a cross-over design (35–50-day washout period) in recreationally active participants found that 20 g/day of creatine for 5 days reduced the primary amplitude of the VO_2_ response and end-exercise VO_2_, in addition to reduced blood lactate compared to placebo. In contrast, others have found no effects on blood lactate and oxygen consumption [39] or fuel utilization [40] when using similar dosing protocols and methodology.

In a recent systematic review and meta-analysis, creatine supplementation during graded exercise tests improved ventilatory threshold (effect size = 0.66, 95% CI: 0.23 to 1.1, *p* = 0.003), had no effect on relative VO_2_max (mL · kg^−1^·min^−1^; effect size = −0.18, 95% CI: −0.45 to 0.09), time to exhaustion (effect size = −0.12, 95% CI: −0.52 to 0.28), or maximal power output (effect size = −0.10, 95% CI: −1.01 to 0.81) [7], but did affect absolute VO_2_max (L · min^−1^) decreasing by an effect size of−0.20 (95% CI: −0.039 to−0.001, *p* = 0.049) compared to placebo. Importantly, this systematic review only evaluated physiological and cardiorespiratory parameters during graded exercise tests, and thus was unable to explore the effects of creatine on surges or finishing sprints.

Furthermore, there is also individual differences and responses to creatine supplementation which may be associated with genetics [41], diet or fiber-type distribution [7,42]. For example, Syrotuik and Bell [42] found that responders to creatine supplementation had a greater proportion of fast twitch (Type II) muscle fibers (responders had 63.1% and non-responders had 39.5%) and had a lower baseline skeletal muscle creatine content (which may be associated with diet [7]).

Animal studies appear to corroborate human studies. For example, Brannon et al. [43] examined 4 weeks of creatine supplementation (3.3 mg · g^−1^ of diet) with and without exercise in rats. Creatine supplementation elevated citrate synthase, a mitochondrial enzyme, and surrogate marker of mitochondrial content, in both conditions. Liu et al. [44] demonstrated that PCr integrates with membrane phospholipids and is important to maintain the integrity and stability of mitochondrial membranes, essential to maintain ATP homeostasis through regulating mitochondrial respiration. Furthermore, creatine supplementation during prolonged low-frequency chronic stimulation did not impair oxidative adaptations and was able to preserve fast twitch glycolytic muscle (i.e. Type IIb) characteristics in rats [45]. These results are intriguing and may suggest that creatine supplementation during high volume training would help preserve fast twitch capabilities without impairing oxidative adaptations. However, future research in humans is warranted to confirm this hypothesis.

### 2.4. Effects on inflammatory markers and post-exercise recovery

Creatine also has antioxidant capabilities and anti-inflammatory effects following endurance exercise [27,46], which may accelerate post-exercise recovery. Santos et al. [47] found that pre-race creatine supplementation (20 g/day for 5 days) reduced the rise in prostaglandin-E2 (PGE2), tumor necrosis factor-alpha (TNF-alpha), and serum levels of creatine kinase (an indicator of muscle damage) compared to placebo in runners after completing a 30-km time trial. Bassit et al. [48] found a significantly smaller rise in TNF-alpha, interferon-alpha, interleukin 1-beta, and PGE2 in elite athletes supplemented with creatine (20 g/day) for 5 days prior to completing a half-ironman competition compared to placebo. In addition, Deminice et al. [49] showed that creatine supplementation (0.3 g/kg/day for 7 days) attenuated the rise in TNF-alpha and C-reactive protein in young soccer players (mean age: 20 years) but had no effect on oxidative stress. In animal models, Deminice and Jordao [50] reported that creatine supplementation (2% addition to habitual diet) for 28 days decreased markers of oxidative stress (thiobarbituric acid reactive species and total lipid hydroperoxide) following 1 h of swimming in Wistar rats. Whether these reductions in acute post-exercise inflammation lead to chronic endurance performance adaptations over time remains to be elucidated [2]. Thus, creatine in both human and animal models has been shown to be beneficial in enhancing markers of recovery following aerobic activities.

In summary, creatine can affect several mechanisms that have the potential to alter endurance physiology and consequently markers of endurance performance, as shown in Figure 1. Specifically, creatine supplementation has been shown to contribute to a faster regeneration of ATP, influence glycogen resynthesis, calcium kinetics, buffer hydrogen ions, and act as an antioxidant in addition to having anti-inflammatory effects following endurance exercise.

## 3. Creatine supplementation and endurance performance

Despite the large number of studies that have examined the effects of creatine supplementation on resistance training [1,9], there are limited data investigating the effects on endurance exercise performance [1], as shown in Table 1.

**Table 1.** Summary of studies examining creatine supplementation on time to exhaustion or time trial performance.

First Author, Year	Population	Supplement Protocol	Endurance Test	Results
Anomasiri et al. 2004	*N* = 19; Trained swimmers	CR: 10 g/day (split into 2 doses) for 7 days or PLA	400 m swim	CR ↑ final 50 m (i.e. swimmers were faster in the final 50 m).
Balsom et al. 1993	*N* = 18; Well-trained males	CR: 20 g/day for 6 days or PLA	6-km TT; TTE at 120% vVO_2_max	CR ↓ (i.e. slower) TT (23.36 ± 0.82 vs 23.79 ± 0.85 min); TTE ↔
Chwalbinska-Moneta et al. 2003	*N* = 16; Elite male rowers	CR: 20 g/day for 5 days or PLA	GXT TTE and anaerobic capacity test (7 Watts/kg)	CR ↔ on GXT peak power (trend for CR to be superior). CR ↑ power output at LT; CR ↑ TTE during anaerobic test by 12.1 sec
Engelhardt et al. 1998	*N* = 12regionally competitive triathletes	CR: 6 g/day for 5 days or PLA	30-min at 3 mmol/L followed by 5 × 15 sec @ 7.5W/kg with 45 seconds of rest then 120 seconds of rest, then 5 × 15 sec followed by 30 min of aerobic exercise	CR ↑ interval work by 18%; ↔ on aerobic performance; In participants unable to complete the final 30 minutes of exercise at baseline, CR ↑ TTE by 4 min
Jacobs et al. 1997	*N* = 26; Healthy recreational and competitive athletes	CR: 20 g/day for 5 days or PLA	TTE at 125% VO_2_max	CR ↑ TTE (+9%)
McNaughton et al. 1998	*N* = 16males; Elite surf-ski or white-water kayak paddlers	CR: 20 g/day for 5 days or PLA	90 sec, 150 sec, 300 sec tests.	CR ↑ work completed compared to PLA
Prevost et al. 1997	*N* = 18(*n* = 10males, *n* = 8 females); healthy	CR: 18.75 g/day for 5 days and 2.25 g on the 6th day or PLA	TTE at 150% VO2peak continuously, intermittently (60 sec on/120 sec rest or 20 sec on/40 sec rest or 10 sec on/20 sec rest).	CR ↑ work in all trials
Rico-Sanz et al. 2000	*N* = 14trained cyclists	CR: 20 g/day for 5 days or PLA	TTE alternating every 3 min between 30 and 90% MPO	CR ↑ TTE at 90% MPO
Rossiter et al. 1996	*N* = 38(*n* = 28males, *n* = 10 females); Competitive rowers	CR: 0.25 g/kg for 5 days or PLA	1000 m rowing performance	CR ↑ performance (2.3 seconds), PLA ↔
Schaffer et al. 2019	*N* = 11; Recreationally active males	CR: 20 g/day for 5 days followed by 2 g/day	TTF above CP	CR ↑ TTF (11%)
Tomcik et al. 2018	*N* = 18; Well-trained male cyclists (~65 mL/kg/min)	CR: 20 g/day for 5 days followed by 3 g/day for 9 days or PLA with a moderate (6 g CHO/kg) and a high (12 g CHO/kg) diet in a cross over design	120-km time trial interspersed with alternating 1 and 4-km sprints followed by a TTE incline (8%) ride at 90% VO_2_peak.	CR ↑ final sprints. ↔ 120-km and incline TTE
Vandebuerie et al. 1998	*N* = 12; Elite male cyclists	CR: 25 g/day for 4 days or PLA	2.5 hr ride followed by a TTE (at blood lactate of 4 mmol/L) and Sprints (5 × 10 sec)	CR ↑ sprint performance (8–9%); TTE ↔
Van Loon et al. 2003	*N* = 20; Healthy young males	CR: 20 g/day for 5 days followed by 2 g/day for 37 days or PLA	20 min TT and sprints (12 × 12 sec)	CR ↑ supramaximal sprints; ↔ on 20 min TT

CR = creatine; PLA = placebo; TTE = Time to exhaustion; TT = time trial; TTF = time to fatigue; CP = critical power; MPO = maximal power output; VO2max = maximal oxygen consumption; GXT = graded exercise test; LT = lactate threshold; ↓ = significantly reduced or was detrimental; ↑ = significantly increased or was of benefit; ↔ = no effect compared to placebo.

### 3.1. Effects on time to exhaustion and total work completed

The effect of creatine supplementation on time to exhaustion or total work completed might vary according to the protocol and mode of exercise. Balsom et al. [15] found that creatine supplementation had no effect on running time-to-exhaustion at 120% of VO_2_max in well-trained males, an intensity designed to elicit task failure within 3–6 min (creatine: pre = 3.72 ± 0.24 min to post = 3.97 ± 0.25 min; placebo: pre = 3.33 ± 0.20 min to post 3.54 ± 0.30 min). In a similar study with cyclists, Jacobs et al. [51] demonstrated that the time to exhaustion at an intensity of 125% of the participants VO_2_max was significantly improved following creatine supplementation (20 g/day for 5 days), with 13 out of 14 participants showing an average improvement of 11 s. The difference between these studies may be associated with methodological differences (i.e. modality of exercise) and the potential gain in body mass, since creatine supplementation might not be as effective in improving performance in weight-bearing activities, such as running.

Other studies support creatine’s ergogenic abilities to improve time to exhaustion, particularly in shorter duration, higher intensity exercise. When assessing multiple time to exhaustion tests (designed to elicit failure in 600, 330, 180, and 90 s), creatine supplementation increased work completed at all durations, with larger effect sizes for the shorter durations [22]. Furthermore, McNaughton et al. [52] showed significant increases in total work completed in kayakers during 90, 150, and 300-s efforts. Schafer et al. [24] demonstrated that creatine supplementation can increase total work at close to maximal intensities (~97% of peak power output obtained from a graded exercise test) by almost 20 s (205 ± 65 s vs. 184 ± 46 s). Since success in many endurance events is dependent on the athlete’s abilities to exercise at maximal or supramaximal intensities, the results are promising for creatine supplementation to enhance these types of events or races (as highlighted in Figure 2).

Furthermore, when protocols were designed to mimic what might occur in some endurance events, including surges and finishing sprints, the results appear to be more robust. Engelhardt et al. [25] performed an interesting study evaluating interval performance during an endurance session in regionally competitive triathletes before and after creatine supplementation (6 g/day for 5 days). The participants cycled at an intensity equivalent to 3 mmol · L^−1^ of blood lactate for 30 min, immediately followed by ten 15-s intervals (7.5 W/kg for an average of 571 ± 56 W) separated by 45 s of “rest” at the same intensity as during the 30-min effort. Upon completion of the intervals, the participants rested for 120-s prior to re-starting the protocol with another five > 15-s intervals followed by another 30 min of exercise at 3 mmol · L^−1^. Creatine supplementation enhanced the power output achieved during the intervals by 18% and for those participants that were unable to complete the second 30-min bout of exercise during their initial trial, creatine increased their post-interval time to exhaustion by 4 min. In addition, Rico-Sanz et al. [38] performed a protocol whereby participants alternated between 30% and 90% of maximal aerobic power output every 3 min. Total time to exhaustion was significantly increased (from 29.9 ± 3.8 to 36.5 ± 5.7 min) following creatine supplementation (20 g/day for 5 days) in these highly trained athletes. Vandebuerie et al. [53] had elite cyclists complete 150 min of cycling followed by a time to exhaustions at an intensity associated with a blood lactate of 4 mmol · L^−1^. Following the time to exhaustion, participants were asked to complete five >10-s maximal sprints. The time to exhaustion was no different following creatine supplementation; however, peak and mean power output increased by 8–9% for all sprints. More recently, Tomcik et al. [32] found no statistically significant difference during a 120-km time trial in elite cyclists; however, they found improvements in the final 1-km and 4-km sprints. This is of particular interest because even in ultra-endurance events where glycogen stores are depleted and mean exercise intensity is well below the second ventilatory threshold (VT2), one can still benefit from creatine supplementation with enhanced performance in the closing sprints. The authors also investigated the impact of the creatine mediated gain in body mass using a time to exhaustion protocol on an 8% incline at 90% of VO_2_max to determine if the gain in body mass influenced performance (hill climbing). The authors concluded that power output in the closing sprints of an exhaustive time trial was increased following creatine supplementation and the creatine mediated increase in body mass had no detrimental effect on hill climbing performance.

Overall, the results appear promising for creatine to enhance time-to-exhaustion endurance performance, particularly for short-duration exercise (~3 min) or in protocols that mimic real-life race scenarios, where continuous exercise at a lower intensity is interspersed with repeated burst of high-intensity efforts. These benefits may be associated with enhanced glycogen content and enhanced power output due to altered calcium kinetics, as shown in Figure 1.

### 3.2. Effects on time trial performance

While it appears that creatine supplementation can be an effective strategy to enhance time to exhaustion or total work completed, improvements in these types of performance tests do not necessarily translate to time trial performance, which are considered a more valid measure of endurance performance [54]. In contrast to the positive effects of creatine supplementation on time to exhaustion, current research to date has failed to consistently show improvements in time trial performance. For example, Balsom et al. [15] found that creatine (20 g/day for 6 days) impaired 6-km running performance compared to placebo, which the authors noted was likely due to the increase in body mass (+0.9 kg). These performance findings are in contrast to Tomcik [32] but appear to be associated with gains in body mass altering weight-bearing activities (e.g. running) to a greater extent than non-weight bearing activities (e.g. cycling). van Loon et al. [40] found no effect of creatine on a cycling 20-min work test (no effects on time trial performance or main effect of time) and Tomcik et al. (2018) found no effect on mean power output or time to complete a 120-km time trial, although as discussed sprint finish performance was improved.

Creatine supplementation may be more beneficial when exercise is performed at a higher intensity, thus requiring a larger contribution from anaerobic energy production, as shown in Figure 2. Therefore, during continuous, prolonged, steady-state moderate intensity endurance exercise, creatine is unlikely to have a significant benefit. However, in higher intensity races, creatine may be of benefit. For example, Rossiter et al. [55] found improvements in the 1000-m rowing performance (~2.3 s) following creatine supplementation. Upon closer examination, the main differences between groups were in the last two final 200-m splits (i.e. 600–800 m and 800–1000 m splits), highlighting the benefits that creatine might have in events where the end-spurt is a key determinant of performance and supports the research by Anomasiri et al. [33] (previously discussed) that found improvements in the final 50 m of a 400-m swim.

### 3.3. Creatine supplementation and intermittent endurance sports: enhancing high-intensity repeatability?

While time trials are often used to evaluate endurance exercise performance, this type of exercise test does not account for the variable nature of many endurance events (i.e. adjusting to competitors). In different endurance events (e.g. cross-country skiing, mountain biking, cycling, triathlon, etc.), numerous surges in intensity are performed. These may vary depending on the event, the skill level of the athletes, the terrain, race dynamics, course design, and preplanned race tactics. Typically, these surges can be characterized as short-duration (<15 s) such as shown in cycling races performed at power outputs associated with VO_2_max [56–59]. The intensity of these efforts is particularly important, with participants in cross-country mountain biking spending as much as ~40% of their race time at intensities above the second ventilatory threshold [60,61]. In cross-country mountain biking, the number and pattern of surges performed are similar to that observed in team sports (repeated efforts of 5–15 s in duration at intensities above that associated with VO_2_max) [62] than in traditional, steady-state endurance events. As such, being able to withstand numerous surges in intensity during a race, termed “high intensity repeatability” (i.e. the ability to perform numerous bouts of maximal to supramaximal intensity) has been recently proposed as a determinant of endurance performance [63]. While there are no studies that have directly assessed the effects of creatine supplementation on high-intensity repeatability, previous research has confirmed the efficacy of creatine in improving repeated sprint performance during endurance protocols, as shown in Table 1.

### 3.4. Creatine supplementation and critical power

For high-intensity endurance exercise performance, understanding the curvilinear relationship between power output and the time it can be sustained is important [64]. The power output achieved when this relationship levels off is known as the critical power [64]. Once work is performed above the intensity associated with critical power, it is said that the athlete is on “borrowed time” and exhaustion is fast approaching [35]. The amount of work that can be done above critical power is called W’ and is related to muscle PCr content which can be replenished when exercise is performed at low intensities (e.g. below the first ventilatory threshold) [65]. There are a limited number of studies exploring these relationships following creatine supplementation with mixed results. Smith et al. [22] loaded untrained university students with creatine (20 g/day for 5 days) and found significant increases in work capacity but not critical power. These results were replicated by Miura et al. [66] in healthy participants using a cross-over design (with a 6-week wash out period). In contrast, Vanhatalo and Jones [67] found no effect on work capacity or critical power following 20 g/day for 5 days on a 3-min all-out test. Interestingly, Fukuda et al. [68] and Schafer et al. [24] found improvements in performance above critical power due to an increase in W’ in recreationally active participants. Schafer et al. [24] further noted that creatine induces similar magnitudes of neuromuscular fatigue, despite the enhanced supra-critical power performance. The enhancement of high-intensity exercise may be associated with the creatine shuttle in skeletal muscle (i.e. transporting ATP from the mitochondria to sites of utilization; [26]) and/or as a hydrogen ion buffer [69] since PCr hydrolysis consumes hydrogen ions. Furthermore, an increase in mechanical efficiency could also contribute to these improvements [70].

Overall, it appears that the benefits of creatine supplementation are modality specific and dependent on the intensity or changes in intensity in a race (i.e. simulating real-life races). This can be applied to events where terrain requires a surge in intensity (e.g. mountain biking), pack racing mentality (5000-m track race), and self-paced rowing time trials (2000 m). Future research is required to assess if creatine can enhance performance in real-life endurance events, with a particular focus on its potential role in “race-defining” moments.

## 4. Current limitations and future research directions

Despite the promising potential mechanisms following creatine supplementation and the potential benefits (although mixed) of augmenting endurance performance, there are still several research questions that need to be addressed. Research is warranted to confirm whether creatine supplementation can enhance finishing end spurts in other sports beyond swimming (e.g. running, road and mountain biking, cross-country skiing, and rowing) and whether creatine supplementation can enhance in-race changes in pace or surges. Furthermore, it is important to determine whether creatine can preserve fast twitch fiber characteristics during high volume training in humans, as demonstrated in rat models using chronic low-frequency stimulation. It may also be prudent to determine whether a periodized approach to creatine may be useful in a yearly training plan. Perhaps combining creatine during high volume training and at specific points in the training plan where creatine may be of benefit (i.e. developing muscular power or speed) can optimize pace surges and/or finishing sprint performance. Furthermore, it will be important to investigate creatine supplementation co-ingested with carbohydrates to enhance endurance performance, as currently, there is limited data to explore this topic. Other important considerations include whether the loading phase is required or perhaps may be of detriment to performance [14], since creatine loading is known to increase intracellular water retention [71]. It is important to note that longer term supplementation protocols when combined with resistance training do not appear to alter the ratio of intracellular water content relative to skeletal muscle mass [72]. Furthermore, there may be sex-based differences and differences across the menstrual cycle [73]. Moore et al. investigated 20 g/day of creatine for 5 days during the follicular and luteal phases of water retention and body mass changes. There were no changes in intracellular, extracellular, total body water, or body mass during the follicular phase; however, all fluid compartments increased in the luteal phase (despite no changes in body mass). As such, future research may be required to examine the impact of a low-dose creatine strategy without a loading phase on extracellular and intracellular water, body mass, and performance in both male and female endurance athletes. In addition, resistance training is known to enhance the running economy [74,75] and reduce the risk of injury in endurance and team sport athletes [74]. It still remains unclear if creatine supplementation can augment these beneficial effects in endurance athletes. Recent emerging evidence is that creatine can also impact bone strength [76–78], however, whether creatine can impact bone health and reduce the risk of fractures in endurance athletes is yet to be elucidated.

## 5. Potential practical applications/recommendations

Based on the current scientific evidence, the following practical applications and recommendations are provided:

- For non-weight bearing endurance activities, a creatine loading phase of 20 g/day (or 0.3 g/kg/day) separated into four equal proportions for 5-7 days is sufficient to saturate muscle creatine stores. A maintenance dose of 5 g/day (or 0.03 g/kg/day) can be sustained thereafter. For weight-bearing endurance athletes, the loading phase should be avoided due to the potential impact on water retention and gains in body mass. A lower dose of creatine (3–5 g/day) is sufficient to saturate creatine stores (+20%) over a 4-week period.
- Creatine can be added concomitantly as part of an athlete’s training routine where considering aerobic high-intensity intervals are used to enhance aerobic capacity because of creatine’s ability to augment high-intensity performance [19,79].
- Creatine monohydrate is the most researched form of creatine [5,14] and there is a lack of evidence for the efficacy of alternative forms of creatine to be superior to creatine monohydrate. Nearly all of the research investigating creatine on endurance performance has used creatine monohydrate.
- When loading with creatine, it appears beneficial to ingest creatine close to exercise training due to the upregulation of creatine transporters [80,81] and co-ingestion of creatine with carbohydrates appears to be an effective strategy to enhance both the uptake of creatine and for glycogen re-synthesis [30].
- Limited data exist that directly compare males and females in response to creatine supplementation; however, females have higher intramuscular creatine stores and may, therefore, be less responsive to creatine supplementation [7,82–84] and creatine may influence females differently across the menstrual cycle [73].
- Presently, there is a lack of evidence investigating the impact of creatine on endurance performance in children and adolescents [85].
- Creatine supplementation is recommended for sports that include high-intensity burst, multiple surges, or finishing end spurts, such as cross-country skiing, mountain biking, team sports, swimming, rowing, triathlon, and cycling.
- Due to individual variability in response to creatine supplementation, based on initial creatine muscle stores [42], fiber-type distribution [42], and genetics [41], it is advisable that athletes practice with the supplement during the off-season.
- Creatine appears to be beneficial to both recreational and highly trained endurance athletes.

## 6. Conclusion

In summary, there is a growing body of research demonstrating potential mechanisms to support the use of creatine supplementation to enhance endurance performance. Mechanistically, creatine supplementation elevates PCr and glycogen content alters calcium handling and force production, reduces oxidative stress and inflammation, enhances ATP transport from the mitochondria to sites of utilization, increases hydrogen ion buffering, and may possibly influence mitochondrial biogenesis, whole-body oxygen kinetics, and ventilatory threshold. In contrast, creatine causes an increase in body mass, which may be detrimental to endurance performance, especially in weight-bearing sports (e.g. runners). Overall, research on the effects of creatine on endurance performance shows mixed results. However, creatine shows promise to enhance the ability to change pace and to perform a fast-finishing sprint. Future research is warranted to investigate creatine supplementation in a variety of endurance modalities (i.e. non-weight bearing and weight bearing), as well as creatine during high volume endurance training on muscle morphology and physiology.

## Abbreviations

Creatine: CR; placebo: PLA; phosphocreatine: PCr; ATP: adenosine triphosphate; ADP: adenosine diphosphate; W:’ anaerobic work capacity; CHO: carbohydrates; VT1: first ventilatory threshold; VT2: second ventilatory threshold
